# Illness Severity and Outcomes Among Enteric Fever Cases From Bangladesh, Nepal, and Pakistan: Data From the Surveillance for Enteric Fever in Asia Project, 2016–2019

**DOI:** 10.1093/cid/ciaa1320

**Published:** 2020-12-01

**Authors:** Ashley T Longley, Caitlin Hemlock, Kashmira Date, Stephen P Luby, Jason R Andrews, Samir K Saha, Isaac I Bogoch, Mohammad T Yousafzai, Denise O Garrett, Farah N Qamar

**Affiliations:** 1 National Foundation for the Centers for Disease Control and Prevention, Atlanta, Georgia, USA; 2 Global Immunization Division, Centers for Disease Control and Prevention, Atlanta, Georgia, USA; 3 Applied Epidemiology, Sabin Vaccine Institute, Washington, DC, USA; 4 Infectious Diseases and Geographic Medicine, Stanford University, Stanford, California, USA; 5 Child Health Research Foundation, Department of Microbiology, Dhaka Shishu Hospital, Dhaka, Bangladesh; 6 Bangladesh Institute of Child Health, Dhaka Shishu Hospital, Sher-E-Bangla Nagar, Dhaka, Bangladesh; 7 Department of Medicine, University of Toronto, Toronto, Ontario, Canada; 8 Department of Pediatrics and Child Health, Aga Khan University, Karachi, Pakistan

**Keywords:** enteric fever, typhoid, severity, mortality, antimicrobial resistance

## Abstract

**Background:**

Enteric fever can lead to prolonged hospital stays, clinical complications, and death. The Surveillance for Enteric Fever in Asia Project (SEAP), a prospective surveillance study, characterized the burden of enteric fever, including illness severity, in selected settings in Bangladesh, Nepal, and Pakistan. We assessed disease severity, including hospitalization, clinical complications, and death among SEAP participants.

**Methods:**

We analyzed clinical and laboratory data from blood culture–confirmed enteric fever cases enrolled in SEAP hospitals and associated network laboratories from September 2016 to September 2019. We used hospitalization and duration of hospital stay as proxies for severity. We conducted a follow-up interview 6 weeks after enrollment to ascertain final outcomes.

**Results:**

Of the 8705 blood culture-confirmed enteric fever cases enrolled, we identified 6 deaths (case-fatality ratio, .07%; 95% CI, .01–.13%), 2 from Nepal, 4 from Pakistan, and none from Bangladesh. Overall, 1.7% (90/5205) of patients recruited from SEAP hospitals experienced a clinical complication (Bangladesh, 0.6% [18/3032]; Nepal, 2.3% [12/531]; Pakistan, 3.7% [60/1642]). The most identified complications were hepatitis (n = 36), septic shock (n = 22), and pulmonary complications/pneumonia (n = 13). Across countries, 32% (2804/8669) of patients with hospitalization data available were hospitalized (Bangladesh, 27% [1295/4868]; Nepal, 29% [455/1595]; Pakistan, 48% [1054/2206]), with a median hospital stay of 5 days (IQR, 3–7).

**Conclusions:**

While defined clinical complications and deaths were uncommon at the SEAP sites, the high proportion of hospitalizations and prolonged hospital stays highlight illness severity and the need for enteric fever control measures, including the use of typhoid conjugate vaccines.

Enteric fever (typhoid and paratyphoid fevers), caused by *Salmonella enterica* serovars Typhi (*S.* Typhi) and Paratyphi A, B, and C (*S.* Paratyphi), remains an important public health problem in lower-income countries, especially in South Asia and sub-Saharan Africa [1, 2]. Typhoid fever is estimated to cause approximately 11 million illnesses and 116 800 deaths globally, while paratyphoid fever is estimated to cause 3.4 million illnesses and 19 100 deaths [2]. *S.* Typhi is the most common bloodstream infection in South Asia [3], the region that accounts for almost 70% of the global enteric fever burden and mortality [2].

Before the widescale implementation of antibiotics, an estimated 10–15% of patients with enteric fever developed clinical complications, the most severe including intestinal perforation, gastrointestinal bleeding, and neurological complications [4]. Other complications such as hepatitis, pneumonia, myocarditis, and cholecystitis are well described [5]; however, there is a paucity of recent data on the spectrum and frequency of clinical complications [6, 7].

Additionally, determining the case fatality remains difficult for several reasons [7]. Global disease burden and mortality estimations have typically relied on available data from multiple sources; however, methodological differences, varying sample sizes, and the use of different confirmatory tests limit the ability to pool data across various studies. Existing reports of case fatality range from 0% to 23% depending on the geographic location and surveillance method used [6–9]. The most commonly estimated case-fatality ratio is 1% [10], although estimates are higher among those infected with multidrug-resistant strains and differ among age groups and regions [4, 7–9, 11].

Recent data suggest a decline in enteric fever mortality, likely due to improvements in clinical capacity and access to effective treatments in endemic countries [8]. Updated mortality estimates, rates of complications, and severity data are necessary to understand the burden of severe typhoid and inform health policy, especially with growing antimicrobial resistance [7, 12]. The Surveillance for Enteric Fever in Asia Project (SEAP), a multicountry, prospective, population-based surveillance project, was established to characterize the burden of enteric fever in Bangladesh, Nepal, and Pakistan [13]. Here, we describe illness severity, including hospitalization, duration of hospital stays, clinical complications, and deaths, among blood culture–confirmed enteric fever cases over 36 months of surveillance in the SEAP study.

## METHODS

### Study Setting

We conducted clinical and laboratory surveillance for enteric fever from September 2016 to September 2019 at selected hospitals and through enhanced laboratory surveillance at additional participating hospitals and clinics (laboratory network sites) that provide both outpatient and inpatient services. SEAP sites in Dhaka, Bangladesh, included 2 large pediatric hospitals and 3 laboratories (serving both adult and pediatric populations); in Nepal, 1 hospital in Kathmandu and 1 in Kavrepalanchok, plus 8 laboratories; and in Karachi, Pakistan, 2 tertiary hospitals, 3 laboratories, and surgical wards in 2 additional hospitals. Study site selection was described previously; in brief, Bangladesh, Nepal, and Pakistan were selected as countries with a known burden of enteric fever. A review of health facilities and laboratories led to the inclusion of several sites in each country that had existing evidence of enteric fever burden, the microbiological infrastructure to identify enteric fever by blood culture, inpatient and outpatient facilities, and the ability to obtain population-based sociodemographic data of the facility catchment area [14].

### Definitions

Complications were defined as a diagnosis of a comorbid condition confirmed by the treating physician as a complication of enteric fever. Hospital admission and duration of hospital stay were used as proxies for disease severity. We defined a hospitalization day as one that included an overnight stay. Fluoroquinolone resistance included reduced susceptibility to ciprofloxacin, ofloxacin, levofloxacin, moxifloxacin, and gatifloxacin. Third-generation cephalosporin resistance included resistance to ceftriaxone, cefixime, cefotaxime, cefpodoxime, and ceftazidime. Extensively drug-resistant (XDR) *S.* Typhi is characterized as resistant to first-line antibiotics (chloramphenicol, ampicillin, and trimethoprim-sulfamethoxazole), fluoroquinolones, and third-generation cephalosporins [12].

### Inclusion Criteria

We selected patients with blood culture–confirmed Typhi or Paratyphi A of any age who (1) presented to outpatient departments, were identified in inpatient/surgical wards, or identified through hospital laboratories at SEAP hospitals or (2) were identified at laboratory network sites. Inclusion criteria by enrollment location are described in Table 1.

**Table 1.** Inclusion Criteria by Enrollment Location for the Surveillance for Enteric Fever in Asia Project (SEAP)

Enrollment Location	Inclusion Criteria
Outpatient department	A patient presenting to the outpatient department (including emergency department) with a self-reported history of fever for ≥3 consecutive days within the last 7 days and living within the defined catchment area
Inpatient department	A patient admitted to the hospital with a clinical suspicion or a confirmed diagnosis of enteric fever at any time during hospitalization
Hospital laboratory	A patient with blood culture–confirmed *Salmonella* Typhi or Paratyphi A
Surgical department	A patient with a nontraumatic ileal perforation, even in the absence of laboratory confirmation, with no other known etiology
Laboratory network sites	A patient with blood culture–confirmed *Salmonella* Typhi or Paratyphi A

### Data Collection

Study teams reviewed the medical charts of all participants from SEAP hospitals at the time of the enrollment visit and collected data on patient age, medications prescribed, duration of hospital admission for those hospitalized, complications, and clinical outcomes. Medical charts from laboratory network patients were not available for review. To ascertain final patient outcomes (eg, resolved, died), study teams attempted to contact all patients with blood culture–confirmed enteric fever enrolled in the study by telephone by calling up to 5 times over a 2-week period beginning 6 weeks following enrollment. The study teams also collected phone numbers of family members to contact if the patient could not be reached directly. If a patient sought medical care related to his or her illness during the 6 weeks between enrollment and follow-up, study teams attempted to obtain medical records from those visits to perform chart reviews.

### Statistical Analysis

We calculated frequencies of complications and outcomes. We conducted bivariate comparisons using Pearson’s chi-square test and used the Wilcoxon rank-sum test for assessing the differences in the median values of continuous variables.

Country-specific univariable and multivariable logistic regression models were used to identify potential predictors of hospitalization among cases identified from the SEAP sites, including age, gender, serovar (*S.* Typhi or *S.* Paratyphi A), fluoroquinolone resistance, third-generation cephalosporin resistance, prior antibiotic use, and time in days from fever onset to hospital presentation. Odds ratios (ORs) and 95% confidence intervals (CIs) were estimated from the models.

Similarly, country-specific univariable and multivariable negative binomial regression models were used to identify predictors of the duration of hospital stay among patients hospitalized at the SEAP hospitals, including the same predictors stated above and empiric treatment at hospital presentation. Relative risks and 95% CIs were estimated from the models. For all analyses, a *P* value less than .05 was considered statistically significant.

### Ethical Considerations

Informed consent was obtained from all participants or parents/guardians of participants, as well as verbal assent from participants aged 11–17 years old. The study protocol was approved by the Bangladesh Institute of Child Health Ethical Review Committee, Nepal Health Research Council Ethical Review Board, Pakistan Ethical Review Committee, and Stanford University Institutional Review Board. This project was approved by the Centers for Disease Control and Prevention (CDC) human subjects review as research with CDC not directly engaged, so a full institutional review board approval was not required.

## RESULTS

A total of 34 748 patients were enrolled from SEAP sites in Bangladesh, Nepal, and Pakistan, of whom 8705 (25%) were blood culture–confirmed for enteric fever; 5215 (60%) cases were identified from the SEAP hospitals and 3490 (40%) cases were identified through laboratory network sites. Of the confirmed cases, 7591 (87%) were positive for *S*. Typhi and 1114 (13%) were positive for *S*. Paratyphi A (Table 2). There was no laboratory evidence of bacterial coinfection among the blood culture–confirmed cases.

**Table 2.** Characteristics of Patients Enrolled in the Surveillance for Enteric Fever in Asia Project (SEAP)—Bangladesh, Nepal, and Pakistan, 2016–2019

	Bangladesh	Nepal	Pakistan	Total
Patients enrolled, n	17 441	7215	10 092	34 748
Blood culture–confirmed enteric fever^a^ cases, n (%)	4873 (28)	1602 (22)	2230 (22)	8705 (25)
Patients identified from SEAP hospitals,^b^ n (%)	3032 (62)	534 (33)	1649 (74)	5215 (60)
*S.* Typhi, n (% of culture-confirmed cases)	4131 (85)	1367 (85)	2093 (94)	7591 (87)
Age in years, median (IQR)	6 (3–14)	20 (15–25)	6 (3–13)	8 (4–19)
Male gender, n (%)	2754 (57)	942 (59)	1286 (58)	4982 (57)
Days from fever onset to hospital presentation, median (IQR)	5 (3–6)	4 (3–7)	6 (4–10)	5 (3–7)
Antibiotic use prior to enrollment visit, n (%)	1651 (34)	390 (24)	1292 (58)	3333 (38)
Hospitalization data available, n (%)	4868 (>99)	1595 (>99)	2206 (99)	8669 (>99)
Hospitalized, n (%)	1295 (27)	455 (29)	1054 (48)	2804 (32)
Age in years, median (IQR)	5 (3–10)	19 (14–24)	6 (3–13)	7 (3–16)
Male gender, n (%)	733 (57)	238 (52)	618 (59)	1589 (57)
Days from fever onset to hospital presentation, median (IQR)	6 (4–7)	5 (4–8)	8 (5–13)	6 (4–9)
Antibiotic use prior to enrollment visit, n (%)	561 (43)	141 (31)	689 (65)	1391 (50)
Duration of hospital admission in days, median (IQR)	6 (5–9)	6 (4–8)	3 (2–5)	5 (3–7)
*S.* Typhi infection, median (IQR)	7 (5–9)	6 (4–8)	3 (2–5)	5 (3–7)
*S.* Paratyphi A infection, median (IQR)	6 (5–8)	4 (4–6)	3 (2–3)	5 (3–7)
Participated in follow-up interview, n (% of culture-confirmed cases)	4562 (94)	1498 (94)	2029 (91)	8089 (93)
Additional symptoms reported, n (%)	346 (8)	265 (18)	105 (5)	716 (9)
Sought additional medical care, n (%)	251 (73)	135 (51)	69 (66)	455 (64)

N = 34 748.

Abbreviation: IQR, interquartile range.

^a^ *Salmonella* Typhi and Paratyphi A.

^b^Not including laboratory network facilities.

### Clinical Complications

Over 99% (5205/5215) of the patients identified from SEAP hospitals had medical records available for review, of whom 90 of 5205 (1.7%) experienced at least 1 complication during the enrollment visit (Bangladesh, 0.6 % [18/3032]; Nepal, 2.3% [12/531]; Pakistan, 3.7% [60/1642]). Seventeen (19%) patients developed more than 1 complication. Complications included hepatitis (n = 36), septic shock (n = 22), pulmonary complications/pneumonia (n = 13), encephalopathy (n = 12), intestinal perforation (n = 10), gastrointestinal bleeding (n = 9), intestinal obstruction (n = 6), renal impairment (n = 1), and myocarditis (n = 1). Intestinal obstructions were secondary complications only among patients who experienced an intestinal perforation. Patients infected with *S.* Typhi who reportedly consumed antibiotics before hospitalization and those who took a longer time to seek care were more likely to be diagnosed with a complication (Table 3).

**Table 3.** Characteristics of Patients With Blood Culture–Confirmed Enteric Fever (*Salmonella* Typhi and Paratyphi A) Enrolled in the Surveillance for Enteric Fever in Asia Project (SEAP) With Clinical Complications Documented in the Medical Records—Bangladesh, Nepal, and Pakistan, 2016–2019

	Patients with Complications, by Country			All Countries		
	Bangladesh (n = 18)	Nepal (n = 12)	Pakistan (n = 60)	With Complications (n = 90)	Without Complications (n = 5115)	*P*
Demographics						
Age, median (IQR), years	5 (3–8)	19 (14–25)	9 (3–17)	9 (4–17)	6 (3–10)	<.001
Male gender, n (%)	8 (44)	8 (67)	36 (60)	52 (58)	2880 (56)	.78
Isolate characteristics						
*S.* Typhi serovar, n (%)	17 (94)	12 (100)	58 (97)	87 (97)	4516 (88)	.01
Fluoroquinolone resistance, n (%)	18 (100)	8 (67)	60 (100)	86 (96)	4902 (96)	.82
*S.* Typhi third-generation cephalosporin resistance,^a^ n (%)	46 (79)	0 (0)	0 (0)	46 (79)	932 (63)	.01
Severity						
Time from fever onset to hospital presentation, median (IQR), days	6 (3–8)	5 (3–8)	10 (7–16)	8 (5–14)	5 (3–7)	<.001
Prior antibiotic use,^b^ n (%)	8 (44)	8 (67)	53 (88)	69 (77)	2679 (52)	<.001

N = 5205.

Abbreviation: IQR, interquartile range.

^a^Pakistan only, where antibiotic resistance testing results were available: n = 58 with complication, n = 1470 without complications; no third-generation cephalosporin resistance was detected in Bangladesh or Nepal.

^b^Patient or caregiver reported.

### Six-Week Follow-up

Of the 8705 confirmed cases, 3 died during hospitalization. Of the remaining 8702 patients, 8092 (93%) patients or patient caregivers participated in the follow-up interview at 6 weeks: Bangladesh, 94% (4562/4873); Nepal, 94% (1498/1602); and Pakistan, 91% (2029/2230). Study teams were unable to interview 610 (7%) patients; 9 refused to participate and the others could not be reached. In Pakistan, 64% of patients with typhoid with known outcomes were infected with strains resistant to third-generation cephalosporins, as were 71% of those lost to follow-up and 100% of the fatal cases (Table 4).

**Table 4.** Characteristics of Patients With Blood Culture–Confirmed Enteric Fever (*Salmonella* Typhi and Paratyphi A) Enrolled in the Surveillance for Enteric Fever in Asia Project (SEAP) by Outcome 6 Weeks Following Enrollment—Bangladesh, Nepal, and Pakistan, 2016–2019

	Known Outcome^a^ (n = 8089)	Lost to Follow-up (n = 610)	Died (n = 6)
Demographics			
Age, median (IQR), years	8 (4–19)	6 (3–12)	6 (2–33)
Male gender, n (%)	4622 (57)	356 (58)	4 (67)
Isolate characteristics			
*S.* Typhi serovar, n (%)	7037 (87)	548 (90)	6 (100)
Fluoroquinolone resistance,^b^ n/N (%)	7630/7990 (95)	561/602 (93)	6 (100)
*S.* Typhi third-generation cephalosporin resistance,^b^ n/N (%)			
Bangladesh	0/3797 (0)	0/268 (0)	0/0 (0)
Nepal	0/1268 (0)	0/80 (0)	0/2 (0)
Pakistan	1223/1900 (64)	136/192 (71)	4/4 (100)
Severity			
Time from fever onset to hospital presentation,^c^ median (IQR), days	5 (3–7)	5 (3–7)	8 (4–14)
Prior antibiotic use,^d^ n/N (%)	3077 (38)	253 (41)	3 (50)
Hospitalized, n (%)	2600/8056 (32)	199/607 (33)	5 (83)
Duration of hospital stay,^e^ median (IQR), days	5 (3–7)	6 (3–8)	7 (5–9)
Complications,^e^ n (%)	81/4732 (1.7)	8/469 (1.7)	1 (17)

N = 8705.

Abbreviation: IQR, interquartile range.

^a^Does not include the 6 patients who died.

^b^Patients with antimicrobial resistance testing results available.

^c^Includes patients from SEAP hospitals only (not laboratory network sites).

^d^Patient or caregiver reported.

^e^Among patients with medical charts available for review.

A total of 9% (716/8092) of participants contacted reported symptoms following the enrollment visit; 64% (455/716) sought additional medical care (Table 2), and 69% (313/455) of those patients had medical records available for review. One patient was diagnosed with an intestinal perforation 1 day following the hospital outpatient visit, an 18-year-old male from Pakistan with XDR typhoid.

### Case-fatality Ratio

Of the 8095 participants with known outcomes (including the 3 who died during hospitalization), we identified 6 deaths, all culture-positive for *S*. Typhi (overall case-fatality ratio of .07%; 95% CI, .01–.13%). Two fatal cases occurred in Nepal, both adult males (33 and 65 years old) who presented to laboratory network sites and reportedly died after hospital discharge. Four fatal cases occurred in Pakistan, 2 male and 2 female patients (8 months–8 years old). Three of these patients died at the SEAP hospitals during hospitalization and 1 died after the enrollment visit. No deaths were identified in Bangladesh. None of the fatal cases included a diagnosis of intestinal perforations. In Pakistan, 3 patients who died reportedly consumed antibiotics prior to enrollment. All isolates from fatal cases were resistant to fluoroquinolones, and those in Pakistan were all XDR.

Hospitalization data were available for 8062 of 8095 patients with known outcomes. Five of the 2605 hospitalized patients died (case-fatality ratio of .19%; 95% CI, .02–.36%). One patient enrolled as an outpatient (1/5457) died (case-fatality ratio of .02%; 95% CI, 0–.5%).

### Hospitalization

**Figure 1.**
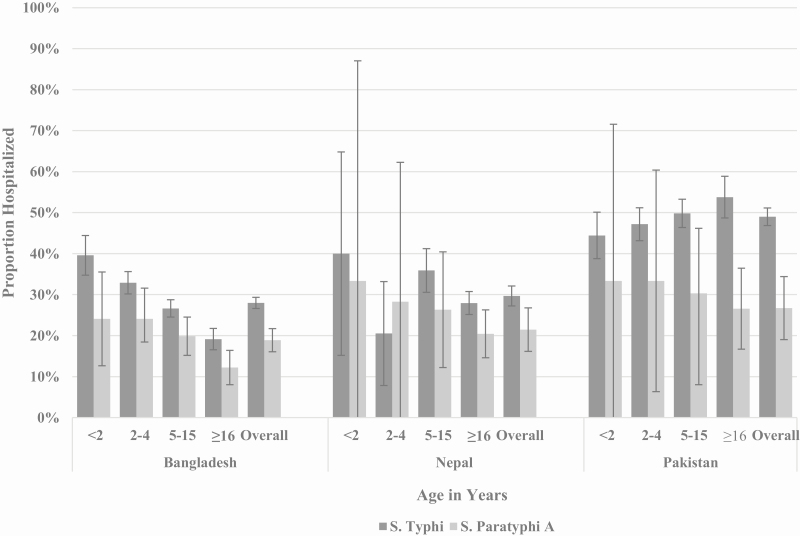
Proportion of *Salmonella* Typhi and *Salmonella* Paratyphi A case-patients enrolled in SEAP who were hospitalized by age in years and by country—Bangladesh, Nepal, and Pakistan, September 2016–September 2019 (*S*. Typhi, n = 7564; *S.* Paratyphi A, n = 1105). Abbreviation: SEAP, Surveillance for Enteric Fever in Asia Project.

Of the 8669 patients with confirmed enteric fever with available hospitalization data, 2804 (32%) were hospitalized. Overall, 2576 (92%) of the hospitalized patients with confirmed enteric fever were infected with *S*. Typhi (Bangladesh, 89% [1155/1295]; Nepal, 89% [404/455]; Pakistan, 96% [1017/1054]). Among patients infected with *S*. Typhi, the highest proportion of hospitalizations occurred among children younger than 2 years old in Bangladesh and Nepal and among those 16 years or older in Pakistan. Across countries, the highest proportion of hospitalizations among those with *S.* Paratyphi A infection was among children younger than 5 years old (Figure 1).

Infection with *S*. Typhi, reported prior antibiotic use, and delayed care-seeking were significant common predictors of hospitalization across countries in the univariable models. In the multivariable models, there was a consistent trend in the direction of the effect for Typhi infection and prior antibiotic use, which was statistically significant in 2 of the 3 countries. Resistance to fluoroquinolones and third-generation cephalosporins was associated with higher odds of hospitalization in Pakistan (Table 5).

**Table 5.** Univariable and Multivariable Analysis of Factors Associated With Hospitalization Among Patients With Blood Culture–Confirmed Enteric Fever (*Salmonella* Typhi and Paratyphi A) Enrolled in the Surveillance for Enteric Fever in Asia Project (SEAP), by Country—Bangladesh, Nepal, and Pakistan, September 2016–September 2019

	Bangladesh		Nepal		Pakistan	
	Univariable (n = 4868)	Multivariable^a^ (n = 3003)	Univariable (n = 1595)	Multivariable^a^ (n = 519)	Univariable (n = 2206	Multivariable^a^ (n = 1548)
Age in years						
<2	2.82 (2.21, 3.60)*	5.51 (1.90, 16.01)*	1.74 (.67, 4.53)	3.19 (.41, 24.96)	.83 (.62, 1.11)	.70 (.48, 1.03)
2–4	2.17 (1.80, 2.64)*	3.87 (1.35, 11.10)*	.76 (.37, 1.55)	2.31 (.75, 7.15)	.92 (.72, 1.17)	.73 (.53, 1.02)
5–15	1.61 (1.34, 1.93)*	3.10 (1.08, 8.90)*	1.47 (1.14, 1.89)*	3.10 (2.01, 4.80)*	1.00 (.80, 1.26)	.99 (.73, 1.33)
≥16	Ref	Ref	Ref	Ref	Ref	Ref
Gender						
Female	Ref	Ref	Ref	Ref	Ref	Ref
Male	1.00 (.88, 1.14)	.91 (.78, 1.07)	.70 (.55, .86)*	.78 (.51, 1.28)	1.09 (.92, 1.29)	1.16 (.93, 1.45)
Species						
*S.* Paratyphi A	Ref	Ref	Ref	Ref	Ref	Ref
*S.* Typhi	1.67 (1.37, 2.03)*	1.43 (1.10, 1.84)*	1.51 (1.09, 2.11)*	1.58 (.91, 2.74)	2.41 (1.63, 3.56)*	1.70 (1.05, 2.74)*
Fluoroquinolone sensitivity						
Susceptible	Ref	Ref	Ref	Ref	Ref	Ref
Reduced susceptibility	1.57 (.93, 2.63)	2.64 (1.27, 5.48)*	.88 (.64, 1.22)	.84 (.45, 1.59)	2.84 (1.81, 4.47)*	1.98 (1.18, 3.32)*
Third-generation cephalosporin sensitivity^b^						
Susceptible	Ref	Ref	Ref	Ref	Ref	Ref
Resistant	…	…	…	…	2.07 (1.74, 2.47)*	1.78 (1.40, 2.27)*
Prior antibiotic use						
No	Ref	Ref	Ref	Ref	Ref	Ref
Yes	1.75 (1.53, 1.99)*	1.32 (1.12, 1.56)*	1.62 (1.27, 2.07)*	1.33 (.61, 2.00)	1.78 (1.50, 2.11)*	2.05 (1.62, 2.58)*
Days from fever onset to hospital presentation^c^						
<3 days	Ref	Ref	Ref	Ref	Ref	Ref
3–5 days	1.14 (.87, 1.49)	1.11 (.85, 1.46)	1.17 (.67, 2.06)	1.10 (.61, 2.00)	.68 (.42, 1.07)	.61 (.38, .99)*
6–9 days	2.04 (1.55, 2.69)*	1.87 (1.40, 2.47)*	2.86 (1.56, 5.25)*	2.53 (1.32, 4.85)*	1.53 (.97, 2.43)	1.17 (.72, 1.90)
≥10 days	2.70 (1.89, 3.87)*	2.55 (1.76, 3.69)*	2.46 (1.21, 5.01)*	2.31 (1.08, 4.92)*	3.67 (2.27, 5.93)*	2.47 (1.50, 4.14)*

Data are presented as ORs (95% CI). *P < .05.

Abbreviations: CI, confidence interval; OR, odds ratio; Ref, reference.

^a^Multivariable models include patients recruited from SEAP hospitals only.

^b^Cephalosporin resistance only detected in Pakistan.

^c^For SEAP hospital patients only; data not available for laboratory network patients.

Data on duration of hospital stay were available for 96% (1911/1981) of patients admitted to SEAP hospitals (Bangladesh, 939/940; Nepal, 150/150; Pakistan 822/891). The median duration of hospital stay was the same for patients with Typhi and Paratyphi A, 5 days (interquartile range, 3–7 days) (Table 2). There were no common predictors for duration of hospital stay across countries. Third-generation cephalosporin resistance was associated with 48–54% longer hospital stays in Pakistan (Table 6).

**Table 6.** Univariable and Multivariable Analysis of Factors Associated With Duration of Hospital Stay Among Hospitalized Patients With Blood Culture–Confirmed Enteric Fever (*Salmonella* Typhi and Paratyphi A) Enrolled in the Surveillance for Enteric Fever in Asia Project (SEAP), by Country—Bangladesh, Nepal, and Pakistan, September 2016–September 2019

	Bangladesh		Nepal		Pakistan	
	Univariable (n = 939)	Multivariable (n = 927)	Univariable (n = 150)	Multivariable (n = 149)	Univariable (n = 822)	Multivariable (n = 790)
Age in years						
<2	1.00 (.63, 1.58)	1.00 (.63, 1.58)	1.47 (.94, 2.31)	1.61 (.95, 2.73)	.99 (.84, 1.17)	.96 (.82, 1.12)
2–4	.97 (.62, 1.53)	.98 (.62 1.55)	1.52 (1.08, 2.16)*	1.53 (1.09, 2.15)*	1.10 (.97, 1.25)	1.04 (.92, 1.18)
5–10	1.06 (.67, 1.67)	1.06 (.67, 1.67)	1.46 (1.27, 1.68)*	1.46 (1.27, 1.68)*	1.05 (.94, 1.80)	1.05 (.94, 1.17)
≥16	Ref	Ref	Ref	Ref	Ref	Ref
Gender						
Female	Ref	Ref	Ref	Ref	Ref	Ref
Male	1.01 (.95, 1.07)	1.01 (.95, 1.07)	.97 (.84, 1.13)	.91 (.79, 1.04)	1.03 (.94, 1.13)	1.10 (1.00, 1.20)*
Serovar						
*S*. Paratyphi A	Ref	Ref	Ref	Ref	Ref	Ref
*S.* Typhi	1.08 (.98, 1.20)	1.08 (.97, 1.19)	1.30 (1.04, 1.63)*	1.27 (1.03, 1.57)*	1.09 (.87, 1.37)	1.02 (.79, 1.33)
Fluoroquinolone sensitivity						
Susceptible	Ref	Ref	Ref	Ref	Ref	Ref
Reduced susceptibility	.77 (.58, 1.01)	.76 (.57, 1.00^a^)*	.85 (.69, 1.05)	.86 (.71, 1.04)	1.26 (.93, 1.69)	1.00 (.75, 1.34)
Third-generation cephalosporin sensitivity^b^						
Susceptible	Ref	Ref	Ref	Ref	Ref	Ref
Resistant	…	…	…	…	1.48 (1.34, 1.64)*	1.54 (1.39, 1.72)*
Prior antibiotic use						
No	Ref	Ref	Ref	Ref	Ref	Ref
Yes	1.01 (.95, 1.07)	1.01 (.95, 1.07)	1.08 (.93, 1.25)	1.12 (.97, 1.29)	.98 (.88, 1.08)	1.06 (.95, 1.18)
Empirically treated						
No	Ref	Ref	Ref	Ref	Ref	Ref
Yes	.99 (.92, 1.06)	.99 (.92, 1.06)	.89 (.74, 1.06)	1.02 (.86, 1.20)	1.27 (1.15, 1.42)*	1.22 (1.10, 1.35)*
Days from fever onset to hospital presentation						
<3 days	Ref	Ref	Ref	Ref	Ref	Ref
3–5 days	.97 (.87, 1.08)	.97 (.87, 1.09)	.85 (.68, 1.07)	.77 (.62, .96)*	1.06 (.84, 1.33)	.99 (.79, 1.24)
6–9 days	.98 (.88, 1.09)	.98 (.87, 1.09)	.94 (.75, 1.18)	.83 (.66, 1.03)	.94 (.75, 1.18)	.86 (.69, 1.08)
≥10 days	.91 (.79, 1.03)	.89 (.77, 1.02)	.89 (.68, 1.17)	.79 (.62, 1.02)	.96 (.77, 1.21)	.85 (.68, 1.07)

Data are presented as RRs (95% CI). *P < .05.

Abbreviations: CI, confidence interval; Ref, reference; RR, relative risk.

^a^Rounding: .76 (.57, .9985); *P* = .0488.

^b^Cephalosporin resistance only detected in Pakistan.

## Discussion

The SEAP study generated comprehensive data on illness severity and outcomes among a large cohort of blood culture–confirmed cases of enteric fever from high-burden settings in Bangladesh, Nepal, and Pakistan. These data are important for understanding the burden of severe typhoid, and the burden that enteric fever places on the healthcare system. This is particularly relevant in settings like South Asia where many severely ill patients are refused admission due to hospital capacity limitations [15].

Mortality is a critical measure of disease severity and is the main driver of typhoid disease burden estimates. A recent meta-analysis estimated the overall case fatality of enteric fever to be 2.49% [9]. While this is markedly higher than the case-fatality ratio identified from SEAP, several of the studies included in the meta-analysis with higher case-fatality rates utilized small sample sizes or included studies from as early as 1960. The 2017 Global Burden of Disease Study estimated an overall case-fatality rate of 0.95%, with the South Asia–specific estimates closer to 1.1% [2]. The widely invoked case-fatality ratio of 1% was estimated using published data from hospital-based studies and separate community-based studies [10]. Because enteric fever is generally treated as an outpatient disease, estimates derived from inpatient surveillance exclude mild cases not admitted to the hospital and may overestimate the case-fatality ratio [16–19]. However, active surveillance at the community level detects and treats disease early, providing very limited information on uncommon outcomes like severe complications and death [16, 20]. We observed such variation in mortality in the SEAP data, in which the case-fatality ratio among inpatients was almost 10 times higher than that of outpatients.

Alternatives to the tertiary hospitals in the SEAP settings are laboratory network sites, data from which are often not included in hospital-based surveillance. These facilities include community-based private consultation centers, clinics, and secondary hospitals, and constitute an important component of a comprehensive enteric fever surveillance system [19]. By including laboratory network sites alongside our hospital-based surveillance, we were able to more accurately estimate the case-fatality ratio compared with utilizing tertiary hospitals alone. While our overall case-fatality ratio of less than 1% is considerably lower than published estimates, it is comparable to recent findings from Dhaka, Bangladesh, that utilized a similar methodology as SEAP and included patients diagnosed through laboratory network sites [20, 21].

Previous studies have reported higher mortality among patients infected with multidrug-resistant *S*. Typhi [7–9]. With improved clinical services and the use of effective antibiotics, case fatality among blood culture–confirmed enteric fever has reportedly decreased in recent decades [8, 20, 21]. However, with widespread antimicrobial resistance, there is a risk of increased severe outcomes. While we did not detect any cephalosporin resistance in Bangladesh or Nepal [22], a recent molecular surveillance study projected that resistance will develop in the region because there are documented cases in Pakistan and India [12, 23]. All deaths from our population were among patients infected with drug-resistant *S*. Typhi, including XDR typhoid among all deaths in Pakistan, highlighting the threat that antimicrobial resistance poses to the currently low case-fatality ratio.

Similarly, clinical complications attributed to enteric fever by treating physicians were infrequently identified among SEAP patients. Hepatitis, septic shock, and pulmonary complications—the complications we commonly identified—are well documented in the literature [8]. Like the fatal cases, most patients with a complication were infected with organisms resistant to antibiotics commonly prescribed in the region. A study from Vietnam reported complications among an estimated 15% of patients with typhoid and found that clinical complications were associated with intermediate fluoroquinolone resistance during a time when these drugs were widely used to treat enteric fever [24]. Because we observed that patients with third-generation cephalosporin resistance were more likely to develop a typhoid-related complication, we anticipate the rates of severe disease to increase as this class of antimicrobials, including ceftriaxone and cefixime, is commonly prescribed empirically in the SEAP settings [25].

A considerable proportion of blood culture–confirmed enteric fever cases were hospitalized across the 3 countries, especially among children who would benefit from typhoid conjugate vaccines (TCVs), which are safe for children as young as 6 months old [26]. Similar to our findings from SEAP, a surveillance study for enteric fever in Bangladesh identified that almost 41% of patients with blood culture–confirmed enteric fever younger than 2 years old were hospitalized [21]. With the introduction of TCVs into routine immunization and improved access to safe water, food, and sanitation and hygiene facilities, we expect a reduction in typhoid hospitalizations, making hospital beds available for patients requiring inpatient care for other conditions [15].

Antibiotics are widely available without prescription in the communities under SEAP surveillance. We observed that hospitalization was associated with antimicrobial use and resistance, especially in Pakistan, which is currently experiencing an ongoing outbreak of XDR typhoid [12]. Delayed care-seeking and self-reported prior antibiotic use, while only moderately reliable [27], were also associated with higher odds of severe disease, consistent with previous findings [8, 28]. Reasons for delayed care-seeking are unknown; possibly some patients presented to the study sites only after treatment failure, especially in cases of drug resistance [29]. Empiric treatment was also common in our sites [25]. Such treatment, in addition to indiscriminate antibiotic usage in settings already detecting drug-resistant enteric fever, may put patients at risk for poor outcomes due to delays in appropriate therapy [4, 11, 30]. Indeed, empiric treatment in Pakistan was associated with a longer duration of hospital stay, most probably due to ineffective initial treatment. Similarly, patients from Pakistan with evidence of XDR typhoid were admitted for approximately 50% longer than those with non-XDR typhoid. The longer duration of hospital stay may be attributed to morbidity but may also be driven by the need for intravenous carbapenem therapy. Typhoid vaccination could potentially reduce the overprescribing of antibiotics, the subsequent spread of antimicrobial resistance [26], and the overall burden on the healthcare system.

An important limitation to our study is the potential to miss the most severe cases of enteric fever. To ascertain complications, data were abstracted from medical records of blood culture–confirmed patients who sought care at the SEAP hospitals. However, most patients with enteric fever likely seek care at pharmacies or informal care systems and never present to hospitals [31]. We may have missed severe disease and enteric fever deaths among those who did not present to a hospital or who did not obtain diagnostic tests, as these are costly and not always required before treatment [15, 16]. Nevertheless, we were able to review over 5000 medical records of blood culture–confirmed cases. We may have missed deaths among patients lost to follow up, especially patients in Pakistan with XDR typhoid infections who would be more likely to have severe outcomes, including death. While our loss to follow-up was low, our estimated case-fatality ratio is likely biased downwards. Because it may be more difficult to contact families of individuals who died, those reached for follow-up may not be representative of the true case-fatality ratio. However, our hospitalization estimates—an important measure of severity—may be overestimated because we calculated the proportion hospitalized only among those patients with enteric fever presenting to a hospital or laboratory network site, which does not consider the large proportion of cases who seek care at pharmacies or other clinics, especially those with mild disease. Last, blood culture sensitivity is estimated to be approximately 60%—or lower for those with prior antibiotic use [32]. Therefore, we likely missed additional enteric fever cases presenting to our study sites who were not confirmed by blood culture, including patients with enteric fever–related intestinal perforations [33]. Future surveillance should prioritize reducing the loss to follow-up of blood culture–confirmed cases to improve the accuracy of case fatality and severe disease estimates.

### Conclusions

While clinical complications and deaths were rarely identified in our large dataset, the high proportion of hospitalizations and prolonged hospital stays, reflecting substantial costs and morbidity, underscores the benefit of immediate interventions. Case fatality from enteric fever is not a static figure; it varies according to context and time. We found a lower-than-expected case-fatality ratio but noted that extensive drug resistance is associated with more severe disease and morality, and global data demonstrate a continuing trend in the emergence and spread of drug-resistant strains. Thus, continuing to monitor enteric fever mortality is important and can be added to existing hybrid surveillance at a low marginal cost. The World Health Organization’s Strategic Advisory Group of Experts (SAGE) on Immunization has recommended TCV introduction in countries with a high burden of typhoid fever or with a high burden of antimicrobial-resistant *S.* Typhi, both scenarios applicable at the SEAP sites [34]. Patient outcomes, disease severity, and the impact of antimicrobial resistance should drive decision making on enteric fever interventions, including vaccine deployment. With support from Gavi, the Vaccine Alliance to introduce TCVs, more countries will need reliable burden data for vaccine implementation. It is also important to note that there is an identified burden of S. Paratyphi A, which is not covered by the current vaccines, further emphasizing the need to monitor trends in disease burden and severity among patients with enteric fever.

## Notes

***Acknowledgments.*** The authors thank all involved in the Surveillance for Enteric Fever in Asia Project, especially the research staff and study participants.

***Financial support.*** This work was supported by the Bill & Melinda Gates Foundation (grant number OPP1113007, INV-008335) and an agreement (subcontract/subgrant) between the Sabin Vaccine Institute (primary grantee for the Surveillance for Enteric Fever in Asia Project grant from the Bill & Melinda Gates Foundation) and the CDC Foundation.

***Supplement spon*** ***sorship.*** This supplement is sponsored by the Sabin Vaccine Institute and made possible by a grant from the Bill & Melinda Gates Foundation.

***Potential conflicts of interest.*** I. I. B. reports consulting fees from BlueDot, outside the submitted work. S. K. S. reports grants from Child Health Research Foundation. All other authors report no potential conflicts. All authors have submitted the ICMJE Form for Disclosure of Potential Conflicts of Interest. Conflicts that the editors consider relevant to the content of the manuscript have been disclosed.

## References

[1] Mogasale V, Maskery B, Ochiai RL, et al. Burden of typhoid fever in low-income and middle-income countries: a systematic, literature-based update with risk-factor adjustment. Lancet Glob Health 2014; 2:e570–80. 25304633 10.1016/S2214-109X(14)70301-8

[2] Stanaway JD, Reiner RC, Blacker BF, et al The global burden of typhoid and paratyphoid fevers: a systematic analysis for the Global Burden of Disease Study 2017. Lancet Infect Dis 2019; 19: 369–81. 30792131 10.1016/S1473-3099(18)30685-6 PMC6437314

[3] Deen J, von Seidlein L, Andersen F, Elle N, White NJ, Lubell Y Community-acquired bacterial bloodstream infections in developing countries in south and southeast Asia: a systematic review. Lancet Infect Dis 2012; 12:480–7. 22632186 10.1016/S1473-3099(12)70028-2

[4] Parry CM, Hien TT, Dougan G, White NJ, Farrar JJ Typhoid fever. N Engl J Med 2002; 347:1770–82. 12456854 10.1056/NEJMra020201

[5] Crump JA, Sjölund-Karlsson M, Gordon MA, Parry CM Epidemiology, clinical presentation, laboratory diagnosis, antimicrobial resistance, and antimicrobial management of invasive salmonella infections. Clin Microbiol Rev 2015; 28:901–37. 26180063 10.1128/CMR.00002-15 PMC4503790

[6] Crump JA, Ram PK, Gupta SK, Miller MA, Mintz ED Part I. Analysis of data gaps pertaining to Salmonella enterica serotype Typhi infections in low and medium human development index countries, 1984–2005. Epidemiol Infect 2008; 136: 436–48. 17686194 10.1017/S0950268807009338 PMC2870843

[7] Qamar FN, Azmatullah A, Bhutta ZA Challenges in measuring complications and death due to invasive Salmonella infections. Vaccine 2015; 33(Suppl 3):C16–20. 25921727 10.1016/j.vaccine.2015.03.103

[8] Azmatullah A, Qamar FN, Thaver D, Zaidi AK, Bhutta ZA Systematic review of the global epidemiology, clinical and laboratory profile of enteric fever. J Glob Health 2015; 5:020407. 26649174 10.7189/jogh.05.020407 PMC4672836

[9] Pieters Z, Saad NJ, Antillón M, Pitzer VE, Bilcke J Case fatality rate of enteric fever in endemic countries: a systematic review and meta-analysis. Clin Infect Dis 2018; 67:628–38. 29522159 10.1093/cid/ciy190 PMC6070077

[10] Crump JA, Luby SP, Mintz ED The global burden of typhoid fever. Bull World Health Organ 2004; 82:346–53. 15298225 PMC2622843

[11] Zaki SA, Karande S Multidrug-resistant typhoid fever: a review. J Infect Dev Ctries 2011; 5:324–37. 21628808 10.3855/jidc.1405

[12] Klemm EJ, Shakoor S, Page AJ, et al Emergence of an extensively drug-resistant salmonella enterica Serovar Typhi clone harboring a promiscuous plasmid encoding resistance to fluoroquinolones and third-generation cephalosporins. MBio 2018; 9:e00105–18. 29463654 10.1128/mBio.00105-18 PMC5821095

[13] Carter A, Garrett DO Introducing typhoid conjugate vaccine in South Asia: lessons learned from the surveillance for enteric fever in Asia project. Clin Infect Dis. Manuscript in preparation, in this supplement. 10.1093/cid/ciaa1296 PMC7705880 33258930

[14] Barkume C, Date K, Saha SK, et al. Phase I of the Surveillance for Enteric Fever in Asia Project (SEAP): an overview and lessons learned. J Infect Dis 2018; 218:188–94. 10.1093/infdis/jiy522 PMC6226726 30304505

[15] Saha S, Santosham M, Hussain M, Black RE, Saha SK Rotavirus vaccine will improve child survival by more than just preventing diarrhea: evidence from Bangladesh. Am J Trop Med Hyg 2018; 98:360–3. 29210350 10.4269/ajtmh.17-0586 PMC5929204

[16] Luby SP, Saha S, Andrews JR Towards sustainable public health surveillance for enteric fever. Vaccine 2015; 33(Suppl 3):C3–7. 25912287 10.1016/j.vaccine.2015.02.054

[17] Saha SK, Baqui AH, Hanif M, et al. Typhoid fever in Bangladesh: implications for vaccination policy. Pediatr Infect Dis J 2001; 20:521–4. 11368111 10.1097/00006454-200105000-00010

[18] Vollaard AM, Ali S, van Asten HA, et al. Risk factors for typhoid and paratyphoid fever in Jakarta, Indonesia. JAMA 2004; 291:2607–15. 15173152 10.1001/jama.291.21.2607

[19] Saha S, Islam M, Saha S, et al. Designing comprehensive public health surveillance for enteric fever in endemic countries: importance of including different healthcare facilities. J Infect Dis 2018; 218:227–31. 10.1093/infdis/jiy191 PMC6226780 30060199

[20] Yu AT, Amin N, Rahman MW, Gurley ES, Rahman KM, Luby SP Case-fatality ratio of blood culture-confirmed typhoid fever in Dhaka, Bangladesh. J Infect Dis 2018; 218:222–6. 10.1093/infdis/jiy543 PMC6226771 30304448

[21] Saha S, Islam MS, Sajib MSI, et al. Epidemiology of typhoid and paratyphoid: implications for vaccine policy. Clin Infect Dis 2019; 68:117–23. 10.1093/cid/ciy1124 PMC6405278 30845325

[22] Qamar FN, Saha S Antimicrobial resistance in typhoidal Salmonella from 3 Asian countries: surveillance of enteric fever in Asia (2016–2019). Clin Infect Dis. Manuscript in preparation, in this supplement.

[23] Britto CD, Dyson ZA, Duchene S, et al. Laboratory and molecular surveillance of paediatric typhoidal Salmonella in Nepal: antimicrobial resistance and implications for vaccine policy. PLoS Negl Trop Dis 2018; 12:e0006408. 29684021 10.1371/journal.pntd.0006408 PMC5933809

[24] Parry CM, Thompson C, Vinh H, et al. Risk factors for the development of severe typhoid fever in Vietnam. BMC Infect Dis 2014; 14:73. 24512443 10.1186/1471-2334-14-73 PMC3923984

[25] Hemlock C, Bogoch I Utilization of blood culture in South Asia for the diagnosis and treatment of febrile illness. Clin Infect Dis. Manuscript in preparation, in this supplement. 10.1093/cid/ciaa1322 PMC7705874 33258939

[26] Andrews JR, Baker S, Marks F, et al. Typhoid conjugate vaccines: a new tool in the fight against antimicrobial resistance. Lancet Infect Dis 2019; 19:e26–30. 30170987 10.1016/S1473-3099(18)30350-5

[27] Vaidya K, Andrews J Antibiotic use prior to hospital presentation among individuals with suspected enteric fever in Nepal, Bangladesh, and Pakistan. Clin Infect Dis. Manuscript in preparation, in this supplement. 10.1093/cid/ciaa1333 PMC7705873 33258935

[28] Bhutta ZA Impact of age and drug resistance on mortality in typhoid fever. Arch Dis Child 1996; 75:214–7. 8976660 10.1136/adc.75.3.214 PMC1511710

[29] Bhutta ZA, Naqvi SH, Razzaq RA, Farooqui BJ Multidrug-resistant typhoid in children: presentation and clinical features. Rev Infect Dis 1991; 13:832–6. 1962094 10.1093/clinids/13.5.832

[30] Kadhiravan T, Wig N, Kapil A, Kabra SK, Renuka K, Misra A Clinical outcomes in typhoid fever: adverse impact of infection with nalidixic acid-resistant Salmonella typhi. BMC Infect Dis 2005; 5:37. 15904505 10.1186/1471-2334-5-37 PMC1164413

[31] Andrews J, Qamar FN Healthcare utilization patterns for acute febrile illness in Bangladesh, Nepal, and Pakistan: Surveillance for Enteric Fever in Asia Project. Clin Infect Dis. Manuscript in preparation, in this supplement. 10.1093/cid/ciaa1321 PMC7705868 33258937

[32] Antillon M, Saad NJ, Baker S, Pollard AJ, Pitzer VE The relationship between blood sample volume and diagnostic sensitivity of blood culture for typhoid and paratyphoid fever: a systematic review and meta-analysis. J Infect Dis 2018; 218:255–67. 10.1093/infdis/jiy471 PMC6226661 30307563

[33] Qazi SH, Qamar FN Healthcare utilization patterns for acute febrile illness in Bangladesh, Nepal and Pakistan: Surveillance for Enteric Fever in Asia Project. Clin Infect Dis. Manuscript in preparation, in this supplement. 10.1093/cid/ciaa1321 PMC7705868 33258937

[34] Strategic Advisory Group of Experts in Immunization. Summary of the October 2017 meeting of the Strategic Advisory Group of Experts in Immunization. Available at: https://www.who.int/immunization/policy/sage/SAGE_oct_2017_meeting_summary.pdf?ua=1. Accessed 16 October 2019.

